# Early Perceptions of COVID-19 Contact Tracing Apps in German-Speaking Countries: Comparative Mixed Methods Study

**DOI:** 10.2196/25525

**Published:** 2021-02-08

**Authors:** Bettina Maria Zimmermann, Amelia Fiske, Barbara Prainsack, Nora Hangel, Stuart McLennan, Alena Buyx

**Affiliations:** 1 Institute of History and Ethics in Medicine Technical University Munich Munich Germany; 2 Institute for Biomedical Ethics University of Basel Basel Switzerland; 3 Department of Political Science University of Vienna Vienna Austria

**Keywords:** COVID-19, contact tracing, app, interview study, newspaper content analysis, privacy paradox, digital surveillance, trust, content analysis, surveillance, privacy, interview

## Abstract

**Background:**

The main German-speaking countries (Germany, Austria, and Switzerland) have implemented digital contact tracing apps to assist the authorities with COVID-19 containment strategies. Low user rates for these apps can affect contact tracing and, thus, its usefulness in controlling the spread of the novel coronavirus.

**Objective:**

This study aimed to assess the early perceptions of people living in the German-speaking countries and compare them with the frames portrayed in the newspapers during the first wave of the COVID-19 pandemic.

**Methods:**

We conducted qualitative interviews with 159 participants of the SolPan project. Of those, 110 participants discussed contact tracing apps and were included in this study. We analyzed articles regarding contact tracing apps from 12 newspapers in the German-speaking countries.

**Results:**

Study participants perceived and newspaper coverage in all German-speaking countries framed contact tracing apps as governmental surveillance tools and embedded them in a broader context of technological surveillance. Participants identified trust in authorities, respect of individual privacy, voluntariness, and temporary use of contact tracing apps as prerequisites for democratic compatibility. Newspapers commonly referenced the use of such apps in Asian countries, emphasizing the differences in privacy regulation among these countries.

**Conclusions:**

The uptake of digital contact tracing apps in German-speaking countries may be undermined due to privacy risks that are not compensated by potential benefits and are rooted in a deeper skepticism towards digital tools. When authorities plan to implement new digital tools and practices in the future, they should be very transparent and proactive in communicating their objectives and the role of the technology—and how it differs from other, possibly similar, tools. It is also important to publicly address ethical, legal, and social issues related to such technologies prior to their launch.

## Introduction

**Figure 1:**
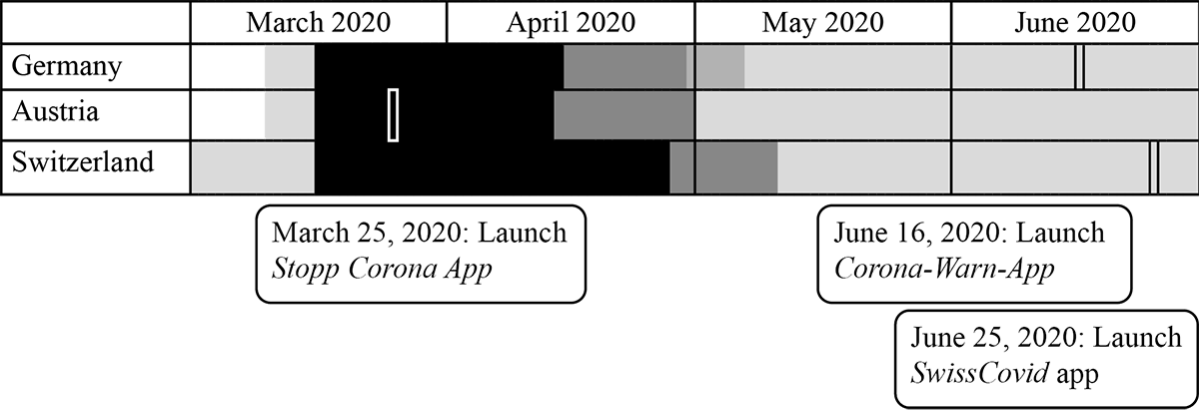
Comparative timeline of COVID-19–related restrictions and contact tracing smartphone apps released from March to June 2020. Black: lockdown period; grey: step-wise easing of restrictions.

During the COVID-19 pandemic, several country-specific contact tracing apps were introduced to assist and supplement analog contact-tracing. In Austria, Red Cross launched the Stopp Corona app on March 25, 2020; Germany launched the Corona-Warn-App on June 16, 2020; and Switzerland launched the SwissCovid app on June 25, 2020 (Figure 1). All these free apps use Bluetooth Low Energy technology to measure the distance and the duration of contact between smartphones that have the app installed. If the tracking function is enabled, random encrypted codes are automatically exchanged and directly saved on the devices whenever a person encounters another user. These random codes do not reveal any names or identities of people or exact locations, and the codes are erased from the smartphones after 14 days. If a person using the app tests positive for COVID-19, they can voluntarily choose to make their random codes anonymously available so that other users who may have come into contact with the infected person can be notified and asked to contact their local health authorities for further instructions.

Although these apps have the same basic functions, there are some important differences among them. First, there are differences concerning the institution that launched the apps—the Swiss and German contact tracing apps were launched by the respective federal governments, whereas the Austrian app was launched by Red Cross Austria, a national nongovernmental organization. Second, the apps differ in the way in which users can report infection. For instance, German app users need to call a hotline after having received a positive COVID-19 test result [1]. Swiss app users receive a code from health authorities that needs to be entered into the app [2]. Austrian app users can alert their contacts directly without contacting any authority, but they need to register their phone numbers to avoid misuse [3]. The Austrian app also includes a symptom checker that allows for a direct alert if symptoms indicative of COVID-19 are entered [3]. Third, there are differences concerning the warning levels: the Swiss and Austrian apps have 2 warning levels (“no warnings” or “potential infection”), whereas the German app has an additional option called “unknown risk,” which is useful in cases wherein the app was not activated long enough to make an assessment. Finally, there are also differences in the management systems used by these apps. In Europe, key management systems for such contact tracing apps include Pan-European Privacy-Preserving Proximity Tracing (PEPP-PT) [4] and Decentralized Privacy-Preserving Proximity Tracing (DP-3T) [5]. PEPP-PT aimed for a centralized data storage system, whereas DP-3T foresaw a decentralized data storage approach on individual phones, thereby aiming to increase data security and privacy [6,7]. The final versions of all 3 apps used the decentralized approach as promoted by DP-3T.

However, it appears that very few people have downloaded and are using these apps. Although surveys conducted in Spring 2020 reported acceptance rates of up to 70% for hypothetical contact tracing apps in Germany and Switzerland [8,9], only 25% and 31% of the total populations in Germany and Switzerland, respectively, had downloaded the app by October 30, 2020, with active user estimations ranging around 21% in both countries [2,10]. On the other hand, only 12% of the population in Austria had downloaded the Stopp Corona App by mid-April 2020 [11,12], and download rates only increased marginally to 12.4% until October 2020 [13]. Modeling studies estimated that for contact tracing apps to be effective, the user rate needed to be at least 56% of the overall population [14] and that uptake rates need to be higher for decentralized data storage systems than for centralized ones [15]. Nevertheless, even lower user rates can have an impact on contact tracing and, consequently, on the ability of the system to control the spread of COVID-19 [16]. Contrary to those estimates, Maccari and Cagno [17] argued that Bluetooth-based, privacy-preserving contact tracing apps are of limited benefit due to potentially high false-positive rates and low sensitivity. Similarly, Rowe et al [18] contended that the French contact tracing app was implemented very quickly without taking into account the available evidence on viral spread. Overall, robust evidence on the actual effectiveness of contact tracing apps is lacking [19].

A large body of the existing literature has assessed users’ perceptions, motivations, and barriers to using mobile health (mHealth) apps, particularly those related to chronic diseases and behavioral interventions [20-22]. Previous research on peoples’ motivation to use mHealth apps suggests that user’s perceived personal value is a crucial motivating factor [23-27], but other factors such as convenient use [26], high quality, trustworthy information provided through the apps [28], and previous experience and training with mobile technology particularly among older adults [27,29] are also mentioned. Stach et al [30] recently suggested a framework to evaluate mHealth apps, including user engagement, app functionality, aesthetics, information quality, therapeutic gain, users’ subjective quality ratings, and perceived impact. Although research on mHealth apps generally focuses on individual usability, including the specific needs of patients with chronic conditions [22,31] or lifestyle support for a healthy diet or physical activity [32,33], contact tracing apps for pandemic control have an additional societal component. Accordingly, in the German context, Trang et al [34] found that appeals to the societal rather than personal benefit of contact tracing apps were most helpful to maximize uptake, particularly if the majority of the population was critical of the apps or undecided about using them. Other survey studies on the willingness to use contact tracing apps indicated that perceived benefit, expected performance, trust in government, and social influence were important motivating factors for using contact tracing apps, whereas privacy concerns were identified as the main hindering factor [8,35-40]. In line with these surveys, Keshet [41] identified a dichotomy in the comments related to Israeli news websites between supporters of contact tracing apps who see them as a protective measure for containment and opponents who see their civil rights violated. Despite issues pertaining to privacy and other civil rights being the most important concern against the use of contact tracing apps [34,42,43], the privacy policies of these apps are not comprehensible by the average individual owing to their complexity [44]. Lack of privacy policies have also been identified for other mobile health apps [45], mHealth apps [46] and privacy concerns, including data safety and confidentiality, are known to negatively affect the uptake of mHealth apps in general [20,46-48].

How digital contact tracing apps are perceived will ultimately be a key factor in people’s willingness to download and use them. Therefore, there is a need to better understand people’s views on the use of digital technology to support COVID-19 contact tracing. The COVID-19 pandemic has been widely covered in the media, mirroring the immense public interest, with traditional mass media, including print and online news media, considered to be one of the preferred information sources [49,50]. The dynamic and uncertain situation at the beginning of the pandemic made people particularly reliant on such information sources. Previous studies have used media content analyses to assess public debates during the COVID-19 pandemic [18,50], as well as other issues that affect population health [51,52]. Some other studies have combined media content analyses with surveys and interviews [53,54].

This study aimed to examine the early perceptions of people living in Germany, Austria, and German-speaking Switzerland toward digital contact tracing apps and compare them with frames as portrayed in the newspapers during the first wave of the COVID-19 pandemic. More specifically, the following research questions were addressed: (1) How did people living in Germany, Austria, and German-speaking Switzerland conceptualize and evaluate digital contact tracing apps during the COVID-19 lockdown in April 2020? (2) How were such apps portrayed in the newspapers, and were there any country-specific differences observed? (3) How did people’s concepts and assessments intersect with the ongoing public debates and policies in April 2020? The countries investigated in this study are quite similar in terms of their culture (eg, language and a strong emphasis on privacy) and have democratic federalist political systems. By comparing data from similar countries, the observed differences can be interpreted in a more precise way [55]. In this context, one relevant difference is the circumstance that Austria had already launched a contact tracing app at the time of the interviews of this study, whereas Switzerland and Germany had not.

## Methods

### Qualitative Interviews

As part of the qualitative, longitudinal, and multinational SolPan (Solidarity in Times of a Pandemic) study [56], qualitative interviews were conducted with 159 individuals in Germany, Austria, and German-speaking Switzerland during the first COVID-19–related lockdown of April 2020. The SolPan Consortium includes 9 European countries (Austria, Belgium, Germany, France, Ireland, Italy, The Netherlands, German-speaking Switzerland, and the United Kingdom). It aims to explore peoples’ experiences during the COVID-19 pandemic, particularly how people describe practices relating to solidarity. Interviews were conducted between April 6 and May 6, 2020, during which country-specific measures were in place to flatten the infection curve. This included various restrictions on movement and contact with other individuals, and the closure of schools, nonessential businesses, and public institutions.

Participants were recruited through online advertisement via university websites, social media networks, convenience sampling, and snowballing. To enable a variety of perspectives, participants were recruited with attention to a range of different demographics, including age, gender, income, household structure, geographic area, education, and employment. Participants received a study information leaflet prior to the interview, and verbal consent was obtained directly before the interview. A researcher-developed interview guide was used to guide the interview, which included a question regarding the use of mobile phones to assist in contact tracing (see Multimedia Appendix 1 for the interview guide). Interviews ranged from 25 minutes to 80 minutes in length; they were conducted online or by telephone and recorded on a digital recorder or using a video chat recorder compliant with the European General Data Protection Regulation. Only audio material was stored. The interviews were transcribed and subsequently pseudonymized. The study was approved by the ethics committees of the Technical University Munich (no. 208/20 S) and the University of Vienna (no. 00544). Interview transcripts were coded by all researchers by using an inductively generated coding scheme developed through the broader SolPan Consortium, using the ATLAS.ti 8.0 software (ATLAS.ti Scientific Software Development GmbH). Coding was checked by a second researcher for consistency. Relevant text passages were extracted using the Atlas.ti query function, analyzed inductively, and summarized in a memo by the first author (BZ), thereby aiming to gain a higher level of abstraction by building concepts and categories. This analysis was performed separately for each country, and 2 authors (SM for Germany and Switzerland, and BP for Austria) double-checked and supplemented the analysis. Then, the concepts and categories were compared between the 3 countries and discussed among the researchers. Interviews were analyzed in German, but memos were written in English. Illustrative quotes were translated from German to English by a native German speaker (BZ) and double-checked by a native English speaker with good German skills (SM).

### Content Analysis of Newspaper Coverage

In parallel to the interview analysis, a newspaper content analysis was conducted to assess what concepts, topics, and concerns were predominant at the time of the interviews across the countries. These insights were used to create a comparative framework analysis for country-specific interpretations and intercountry comparisons. The newspaper content analysis included articles published between March 15 and May 6, 2020, which includes the interview study period, and 3 weeks before the first interviews were conducted.

Three quality newspapers with a national readership and one tabloid from each country were included in the analyses. The selected newspapers are among those with the highest readership and were chosen based on equivalent functions in the respective national media systems [57], to allow for meaningful comparison. Newspapers were included to represent the mass media landscape of public debates for several reasons. First, even though newspapers lose readers to other channels, they still have considerable influence on coverage of other mass media, including social media for long-lasting issues [58]. Moreover, particularly large, high-quality newspapers are still considered a trustworthy source for reliable background information on health-related issues [25]. Finally, the publication format is reliably accessible. Accordingly, the Factiva database (Dow Jones Professional) [59] was used for a systematic search of articles that covered issues related to digital contact tracing. This database was selected because it covered the relevant newspapers, thus allowing for a unified search strategy, and was accessible to the research team. Relevant articles were retrieved with the following search algorithm using full-text search (in German): “(app OR technologie) AND (tracing OR tracking) AND (corona* OR covid-19).” Articles that reported on technology-based tracking or tracing in the context of COVID-19 were included in the study.

A codebook for the media content analysis was developed to collect the following variables: date of publication, medium, importance of topic (ie, contact tracing apps) in the article, country reference, article topics, app evaluation, and stakeholders cited (see Multimedia Appendix 2). The codebook was adapted from a previous study investigating newspaper coverage of medico-scientific issues [51]. Three coders collected these variables; they were trained in 2 online sessions wherein the codebook was discussed and tested in the team and refined upon discussion to make categories exclusive and intersubjectively understandable. For this aspect, 10 articles from each country were double-coded to identify the remaining ambiguities in the codebook. Since all instances of discordant coding could be easily resolved and were assignable to a source of uncertainty that was removed by clarifying the codebook, and because this analysis was used to supplement the qualitative analysis of the interviews, no formal reliability test was conducted. Descriptive statistics were calculated using Excel (Microsoft Corp). To identify key events, the distribution of articles over time as well as information from policy analyses were qualitatively linked to the collected variables. Key events were identified when several newspapers covered the same topic on the same day and/or when a topic was followed-up on several subsequent days.

## Results

In German-speaking countries, both interview participants and newspaper coverage perceived and framed contact tracing apps predominantly as governmental surveillance tools. In the following sections, we first report the findings of the interviews, followed by results of the newspaper content analysis.

### Participants’ Perceptions of Contact Tracing Apps

In the 159 interviews conducted, more than two-thirds (110/159, 69.2%) of the participants commented on contact tracing apps and were included in this analysis (Germany n=29, Austria n=56, and Switzerland n=25). Table 1 presents the demographics of the study participants.

**Table 1:** Demographic distribution of study participants.

Characteristic		Value, n (%)		
		Germany (n=29)	Austria (n=56)	Switzerland (n=25)
**Age (years)**				
	18-30	4 (14)	11 (20)	7 (28)
	31-45	14 (56)	11 (24)	3 (17)
	46-60	4 (36)	20 (59)	6 (40)
	61-70	6 (29)	12 (25)	4 (17)
	>70	1 (3)	2 (3)	5 (17)
**Sex^a^**				
	Female	14 (48)	34 (61)	14 (56)
	Male	15 (52)	22 (39)	11 (44)
**Household**				
	Single	7 (24)	15 (27)	7 (28)
	Couple	10 (34)	20 (36)	8 (32)
	Living with child or children under 12 years	8 (28)	7 (13)	1 (4)
	Living with child or children above 12 years	2 (7)	8 (14)	4 (16)
	Other	2 (7)	6 (11)	5 (20)
**Rural/urban**				
	Big town (eg, capital, >500,000 population)	12 (41)	30 (54)	8 (32)
	Mid-sized or small town	9 (31)	15 (27)	5 (20)
	Rural (eg, village)	8 (28)	11 (20)	12 (48)
**Employment status**				
	Employed (long-term contract)	17 (59)	21 (38)	9 (36)
	Self-employed	2 (7)	9 (16)	3 (12)
	Employed (short-term or precarious contract)	1 (3)	4 (7)	5 (20)
	Unemployed	4 (14)	4 (7)	1 (4)
	Retired	4 (14)	14 (25)	6 (24)
	Other	1 (3)	4 (7)	1 (4)
**Education level**				
	<10 years	0 (0)	5 (9)	8 (32)
	10-14 years (eg, high-school diploma)	9 (31)	18 (32)	3 (12)
	Higher education	20 (69)	33 (59)	14 (56)
**Household net income per month^b^ (before the COVID-19 pandemic)**				
	≤€1400 (US $1693) or ≤CHF 4000 (US $4472)	3 (10)	7 (13)	3 (12)
	€1401-3000 (US $1694-3628) or CHF 4001-7000 (US $4473-7826)	7 (24)	23 (41)	8 (32)
	>€3000 (US $3628) or >CHF 7000 (US $7826)	19 (66)	26 (46)	14 (56)
Total interviews (N=110)		29 (26.3)	56 (50.9)	25 (22.7)

^a^Investigator observed.

^b^Self reported; categories were adjusted based on country-specific income levels.

Most participants stated that they had heard about the option of mobile phone–based contact tracing, but several participants seemed to be uncertain about the function and scope of contact tracing apps or lumped together different technologies. For instance, the concept of individual contact tracing was sometimes confused with population surveillance measures, such as anonymous mobile phone tracking to analyze population behavior during restrictions. These different conceptions of how contact tracing could be employed were also a source of confusion and uncertainty about what mobile phone–based tracing would actually entail.

What I don't quite understand here is whether it´s about who is positive or simply controlling masses of people, such as [navigation systems] in traffic. That uses data to control traffic volume. I don't know exactly how it is supposed to work, that the people who are positive sign up themselves, so to say, or whether the database is maintained and you don't even know whether you are in it or not.

Swiss participant 15

A few participants also stated that they were not well informed about the topic at the time of the interview, indicating that they were not sure whether they could endorse the use of digital tracking tools due to their limited understanding or stating that they were aware of the tools but had not yet formed an opinion on their use.

### Perceiving Contact Tracing Apps as Governmental Surveillance Tools

**Figure 2:**
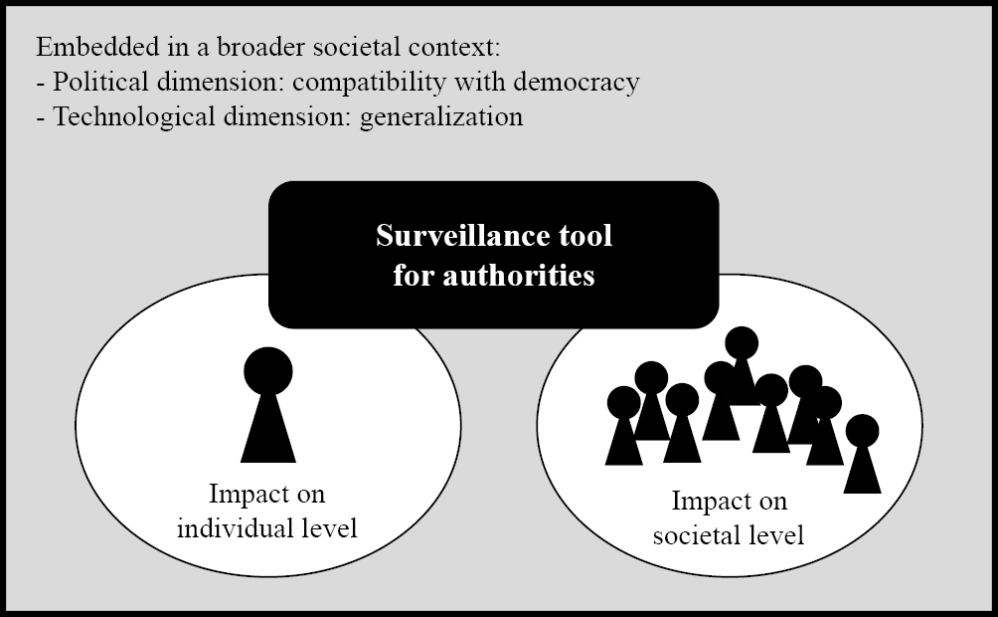
Illustration of interview participants’ perceptions of digital contact tracing apps during the first wave of the COVID-19 pandemic (data collected in April and early May 2020).

Participants perceived contact tracing apps as governmental surveillance tools and embedded them in a broader societal context (Figure 2). However, “surveillance” was framed by participants in different ways. Some participants viewed such surveillance as a mechanism that empowers the authorities to control individual compliance with measures, which provoked negative sentiments. By contrast, other participants justified such contact tracing apps as surveillance tools for authorities that helped contain the pandemic without too many further restrictions. Yet other participants balanced the perils of surveillance against the specific needs to contain the pandemic:

Controlling is not necessarily a threat.

German participant 7

Although German and Swiss participants generally spoke about contract tracing apps only when asked about them specifically, approximately 20% of Austrian participants brought up the topic spontaneously, and their responses were also often more elaborate in length than the responses from Swiss and German participants.

### Compatibility With Democracy

In all three countries, participants related contact tracing apps to Asian countries, such as China, which had already been using digital contact tracing technology for viral containment when the interviews were conducted. Although a few participants referred to the success of Asian countries in virus suppression as proof of the usefulness of these applications, most others used a comparison with “totalitarian states” as an argument against contact tracing apps, stating that the tools were incompatible with democratic values and rights.

Yes, I've heard about [the idea of contact tracing apps] and I completely reject it, I must say because my own data is simply too insecure, not protected and I don't want the state or whoever evaluates this data to know exactly where I was, what I did. And it also has a touch of the Chinese state or something [sic]. In Asia, it is already being practiced without people critically questioning it. But I am absolutely against it.

German participant 41

By contrast, some participants framed contact tracing apps as tools that support authorities to control the viral spread:

I really believe in these apps because I think that if they are handled consistently, they can really reveal a lot of information to the authorities to develop certain measures or to react to the course of the disease [sic].

German participant 3

Participants also indicated a list of prerequisites that they considered crucial for a contact tracing app to be compatible with democracy: trust, privacy, voluntariness, and a time limit on data retention and use. The first prerequisite concerned the level of trust in national and local authorities. Since contact tracing apps were perceived as a surveillance measure of authorities, the level of trust towards these authorities was an important factor for people’s willingness to participate. Participants in all three countries expressed the fear that the pandemic could be exploited by governments to install long-term surveillance systems, weighing the possible benefits of virus containment against the erosion of human rights:

I understand, of course, that this can be used to do contact tracing and potentially contain virus spread and I can see its usefulness. It's just, I'm struggling with the question of how much a crisis justifies intervening in basic human rights? And I think the problem is, if there is no pushback, then you might end up in a situation. I mean, I think that is what happened after the terrorist attacks in London and the US, that suddenly the NSAs of the world are created. And then nobody really knows what kind of information they actually have. And that shouldn't be the case in a democracy. So I think that citizens should be aware that some things are very difficult to undo later on.

German participant 42

The distrust of authorities was expressed most prominently by Austrian participants, who repeatedly called for more transparent communication about the app. They criticized a lack of clarity in communication surrounding how location and movement data were analyzed, possible extensions regarding the functionality of the app, or the role of the Austrian government in an app that was originally launched by Red Cross Austria, a nongovernmental organization. Austrian participants also seemed to be uncertain whether the app could be used for personalized tracking later on. They feared penalties for noncompliance or expressed concerns about a creeping loss of privacy. Others indicated skepticism surrounding a lack of clarity of how data would be used from the app.

So on the one hand the minister says: “Watch out because of this data on Facebook or whatever, or with people that want to call you or steal your login.” On the other hand, he says we should use the Red Cross app. But they don't say what happens to the app or your data in the background [i.e. for other than contract tracing purposes], do they? I don't quite believe them, unfortunately. That’s the only thing I don't really believe, to be honest.

Austrian participant 46

In the second prerequisite, many participants stressed the need to develop contact tracing apps that were compatible with existing privacy and data protection regulations:

I have heard about it [contact tracing apps] and I know that there are different models. So for me, it’s just that I'm generally quite skeptical, that such things could be abused. For example, I don't do anything with YouTube or Facebook or whatever. I'm a bit old-fashioned in that sense. But last night I heard that someone in Switzerland has now created a program where data wouldn't go into any server anywhere, that it would just stay in your own smartphone and one wouldn't know any phone numbers of the people you met. And it wouldn't be stored anywhere. If that were so, I could say, yes, let’s do it.

Swiss participant 31

Third, many participants believed that voluntariness was a key prerequisite for contact tracing apps to be compatible with democratic values and human rights. Although participants in all three countries mentioned this topic, we found it most predominantly mentioned in the Austrian data. Some Austrian participants even envisaged the possibility of “class action lawsuits” [Austrian participant 25] against the government and considered “strategies how to defend” themselves [Austrian participant 27] in case of the possibility of compulsory app use. Two Austrian participants even stated that they would rather die than be monitored by the government.

Finally, participants also thought that these tracing apps should be used temporary and only in the context of special circumstances such as the COVID-19 pandemic. Some participants suggested that the data should be erased when it was no longer needed, whereas others reiterated their concern that the data should only be used for the purposes indicated and not to build a surveillance state. One participant noted that although it made sense to use the app now, they intended to uninstall it by the end of the year, whereas another participant noted that these apps would no longer be justified when vaccines or other treatments are available.

### Embedding Contact Tracing Apps in a General Technological Context

Participants from all countries linked tracing apps to other applications that collect data, such as social media, credit cards, or shopping cards, with the underlying concern that their data might be misused. The idea that others might be able to watch over one’s actions and whereabouts was unnerving for some participants, particularly concerning uncertainty to where the data would be stored, who would have access to data for how long, and whether the crisis would erode privacy principles that would later be hard to re-establish:

I don't know, with technology, I always get the impression that these things creep in like crazy. Like we accept everything with every normal app because we only have the [binary] option not to use it or to accept the terms of use.

Austrian participant 45

Others were specifically concerned about the way the government or other powerful actors might use the information in the future:

We reveal so many things but we don't know yet how this could be used against us in the future […] So people should be careful and I believe that the state has far too much power over us in that context.

Austrian participant 5

Some participants seemed generally suspicious of surveillance technologies because of their potential for abuse, extending their suspicion of platforms such as YouTube or Facebook to digital tracking tools. Others said that their smartphones were already tools for individual surveillance. They suggested leaving smartphones at home to avoid being traced, or jokingly noted that they speak in dialect so that those “listening” will not understand:

I have a Huawei phone and I believe that the data will be delivered to China. That is why I always speak dialect because then the Chinese cannot understand me [laughing] [sic].

Austrian participant 6

Moreover, some participants worried that this technology could negatively affect not only their own lives but also how people relate to each other:

For me, it is not an option for everybody to be so high-tech. That’s what it’s all about. That you can see where somebody is. Who has been with whom in some way and so on. I find that threatening. I find that threatening. And I don't really want that.

Swiss participant 22

Embedding contact tracing apps in their general attitude towards technology led some to generally reject contact tracing apps. For others, by contrast, such consideration relativized their privacy concerns. For instance, one participant noted that they had already been recorded a hundred times on camera when driving into the city. Other participants suggested and expressed acceptance that people had long lost control over their data and their lives.

### Impact on the Societal Level

Some participants framed contact tracing apps as a resource for the common good, stressing their function to help contain the viral spread and protect at-risk individuals. This was particularly pronounced in Germany and Switzerland, whereas only very few Austrian participants articulated this stance. One participant even felt that it was a duty to use the available technology to fight the risk of infection:

But I think that's good, I'm not concerned that [contact tracing apps restrict] freedom and privacy issues, instead I see the benefits. And in South Korea, [digital contact tracing] has really helped to reduce the number of infected people. And I believe that there is a duty to support this.

German participant 35

Others perceived tracing apps as a fast-track back to normality, increasing people’s “freedom to move around” [German participant 26] and as a tool that “makes us all, as a society, more flexible” [Swiss participant 17]. By contrast, a few participants (particularly those in Austria) expressed fears of social pressure, stigma, and panic. One Austrian participant compared the app to the Star of David during the National Socialist Regime that singled out Jewish people. In a similar manner, this participant argued, the app stigmatizes infected people and makes them visible to everyone. It should be noted here that neither of the apps we report on here actually includes the function of making infected individuals visible to others.

### Impact on the Individual Level

Some participants also focused on the impact the app might have on the individual level. Some of them focused on the potential personal benefit when using the app by framing contact tracing apps as a useful individual warning and information tools to proactively avoid COVID-19 infection. They hoped to get warnings to avoid risky situations, thereby decreasing personal uncertainty and providing orientation: “My wife and I, we would download and use it [contact tracing app] immediately and hope to be warned if someone comes too close. Or has come too close” [German participant 12]. This view was mainly taken by participants without practical experience with these apps, as none of the apps include such an immediate warning function. Moreover, contact tracing apps were framed as tools that affect individual responsibility. A few individuals perceived them as a tool that enables them to take individual responsibility: “I would probably also install the app on my cell phone, just because I find it helpful for me to know that if I picked up the virus somewhere, I could inform people, hey, watch out and stuff.” [Austrian participant 15]. One participant said that they wished people would behave more responsibly, indicating that they felt contact tracing apps were only necessary because people were not acting responsibly enough.

Many of those participants who were skeptical of tracing apps were concerned about their privacy, expressing uneasiness about such tools “lying on the bedside table” [German participant 4] and comparing mobile phones to “personal diaries” [German participant 22] that they considered very private. Participants also expressed concerns about data protection in connection with contact tracing apps. To protect their data safely, some advocated for local data storage and against data silos.

### Comparative Content Analysis of Newspaper Coverage

To further contextualize our participants’ perceptions of contact tracing apps, we also performed a content analysis of newspaper coverage on contact tracing apps during the interview period (April 6 to May 6, 2020). By quantitatively and qualitatively comparing coverage from the four most-read newspapers of each country, we identified country-specific differences that laid out the basis for comparative interpretation as presented later in the discussion.

A total of 194 newspaper articles from 12 newspapers of the three German-speaking countries were included in the media content analysis (see Table 2). The nature of newspaper coverage regarding tracing apps was similar in the three countries studied, but Germany tended to have longer articles than the other two countries (see Multimedia Appendix 3).

**Table 2:** Sampling of newspapers and articles.

Country and newspaper			Genre		Publisher		Number of included articles
**Switzerland**							
	Neue Zürcher Zeitung	International quality newspaper		NZZ Mediengruppe		31	
	Tages Anzeiger	National quality newspaper		Tamedia		19	
	Neue Luzerner Zeitung	Regional newspaper		CH Media		10	
	Blick	National tabloid		Ringier		5	
**Germany**							
	Süddeutsche Zeitung	International quality newspaper		Süddeutsche Zeitung GmbH		27	
	Die Welt	International quality newspaper		Axel Springer Verlag		23	
	taz, die tageszeitung	National quality newspaper		Taz Verlags-genossenschaft		16	
	BILD	National tabloid		Axel Springer Verlag		1	
**Austria**							
	Kurier	National quality newspaper		Kurier Zeitungsverlag und Druckerei GmbH		23	
	Der Standard	National quality newspaper		Standard Verlagsgesellschaft m. b. H.		16	
	Die Presse	National quality newspaper		Die Presse Verlags-Gesellschaft m.b.H. & Co KG		23	
	Krone.at	National tabloid		Mediaprint		0	

### Frames and Country-Specific Key Events

From the end of March to the beginning of April, German-language newspapers in all three countries reported geolocation or Wi-Fi–based tracking apps used for the anonymous analysis of population mobility or individual surveillance. Articles reported anonymous mobile phone data analysis to assess population mobility during the lockdown in all three countries. German and Austrian newspapers critically reported political discussions to use nonanonymous data for individual surveillance and raised fundamental concerns regarding basic human rights and democratic legitimacy. Swiss newspapers reported predominantly positive aspects and did not raise such concerns. In Austria, newspapers focused those concerns directly on the Austrian app, which was launched in March. In this context, the president of the first chamber of the Austrian Parliament, Wolfgang Sobotka, speculated publicly about making the Austrian app mandatory in an interview with the Austrian news magazine profil. This fueled already-existing concerns about civil rights and privacy violations in that context.

Similarly, the German health minister Jens Spahn attracted much critical coverage when, on March 25, 2020, he publicly considered including new options for tracking and surveillance in the updated German Epidemic Law. Even though newspapers in Germany reacted with similar concerns as those in Austria, the topic did not stay in the news as long as in Austria. Nevertheless, the launch of Robert Koch-Institut’s “data donation app” on April 9, 2020, was accompanied by predominantly critical coverage about lack of transparency and insufficient data protection.

Starting at the end of March, the Swiss and German newspaper coverage discussed proximity tracing (meaning registering close contacts rather than using location data) as a possible assistance tool for contact tracing after the so-called lockdown period. Even though data protection and privacy issues were discussed, proximity tracing was introduced to potentially overcome these issues. To that end, the German and Swiss newspapers evaluated such applications more positively than Austrian newspapers, which were predominantly critical towards the potential privacy implications of the Austrian app. In mid-April, German and Swiss media picked up disagreements on data storage mechanisms within the PEPP-PT research consortium. Swiss coverage focused on the Swiss epidemiologist Marcel Salathé who argued for a decentralized data storage system. In that context, Swiss newspapers reported supportively about the Swiss contact tracing app in development. In Germany, where researchers had initially favored a centralized data storage system, the same éclat caused discussions about data protection requirements. On April 27, 2020, the German government announced that they had decided on a decentralized data storage system. This was evaluated positively in the newspapers. All three countries also compared nationally discussed solutions of contact tracing apps with international applications, especially those in active use in Israel, China, and South Korea, and criticized those as being largely inconceivable in Western European democracies. Country-specific framings and key events are presented in detail in Multimedia Appendix 4.

### National and International Views on Coverage

In Swiss and German coverage, the international collaboration of researchers involved in research needed for the development of such an app was mentioned more often than in Austria (see Table 3).

**Table 3:** Stakeholders cited in newspaper articles.

Stakeholders cited	Mentions, n (%)		
	Germany (n=67)	Austria (n=62)	Switzerland (n=65)
Governmental actors or politicians	28 (42)	30 (48)	36 (55)
Nongovernmental actors	9 (13)	11 (18)	1 (2)
Scientific and medical experts	22 (33)	5 (8)	32 (49)
Legal experts	4 (6)	18 (29)	9 (14)
Experts from humanities or social sciences	9 (13)	1 (2)	2 (3)
Celebrities or VIPs	1 (1)	1 (2)	0 (0)
Civil society	2 (3)	0 (0)	2 (3)
Private companies	4 (6)	3 (5)	4 (6)
Other	0 (0)	1 (2)	0 (0)

In general, Austrian coverage tended to focus more on a national perspective than Swiss and German coverage, where newspapers repeatedly referred to the international PEPP-PT research initiative. This, in turn, led to coverage on the topic of centralized versus decentralized data storage in these two countries, which did not come up as frequently in Austrian newspaper coverage (see Table 4). In all three countries, tracing and tracking applications were repeatedly connected to totalitarian surveillance states, such as China, or even Asian democracies such as South Korea, where surveillance apps were used as containment strategies.

**Table 4:** Topics mentioned in newspaper articles.

Topic	Germany (n=67)		Austria (n=62)		Switzerland (n=65)	
	Topic total, n (%)	Main topic^a^, n (%)	Topic total, n (%)	Main topic, n (%)	Topic total, n (%)	Main topic, n (%)
GPS motion tracking at population level (anonymized)	3 (4)	2 (3)	4 (6)	0 (0)	16 (25)	6 (9)
GPS motion tracking on an individual level	12 (18)	4 (6)	13 (21)	6 (10)	4 (6)	1 (2)
“Datenspende” app (Robert Koch-Institut)	8 (12)	5 (7)	0 (0)	0 (0)	0 (0)	0 (0)
Development of contact tracing apps	14 (21)	10 (15)	20 (32)	11 (18)	26 (40)	9 (14)
Functioning of contact tracing apps	19 (28)	9 (13)	18 (29)	6 (10)	24 (37)	4 (6)
Centralized vs decentralized data storage	12 (18)	4 (6)	1 (2)	1 (2)	7 (11)	0 (0)
Legal or ethical issues regarding tracing apps	5 (7)	3 (4)	38 (61)	19 (31)	33 (51)	15 (23)
Other relevant topics	4 (6)	4 (6)	0 (0)	0 (0)	0 (0)	0 (0)
Tracking/tracing technology that not the main topic	25 (37)	N/A^b^	17 (27)	N/A	29 (45)	N/A

^a^A topic was defined as the “main topic” if it was mentioned in the title or the first paragraph of the article. There was only one main topic per article but otherwise an indefinite number of topics could be coded if necessary.

^b^Not applicable.

## Discussion

Even though policymakers in Germany, Austria, and Switzerland framed contact tracing apps as safe and helpful tools to contain the viral spread, most participants perceived contact tracing apps as governmental surveillance tools. This perception was represented in all interviews and across all three countries. What varied, however, was how people framed and assessed this form of governmental surveillance. Many participants reflected on the compatibility of contact tracing apps with Western democracies. Newspaper coverage periodically framed these apps in a similar fashion by using three frames that were predominantly covered in April 2020: (1) critical examination of the impact of contact tracing apps on individual privacy and the compatibility with existing data protection regulations; (2) simultaneous framing and comparison with digital surveillance tools used in Asian countries, especially China, provoking a contrasting view to Western democracies; and (3) periodical focus on country-specific political and scientific stakeholders.

Privacy issues were a predominant concern expressed by participants and in newspaper coverage; these issues also mirror the early concerns expressed in the literature [43,60]. People were concerned about the impact of contact tracing apps on individual freedom and privacy, in line with concerns commonly expressed in the newspaper articles reviewed in this study. Participants emphasized that it was a matter of principle for them: The use of a digital tool that was perceived to be so intrusive in people’s privacy should never be made mandatory. Indeed, the voluntariness of contact tracing apps is also debated and emphasized by experts, including what voluntariness means in this context [61-63]. Nevertheless, ethicists have more recently criticized this emphasis on privacy and pointed to other ethical issues that were left aside in app development, such as safety and effectiveness [60,64], social justice issues [65,66], or the potentially problematic policy influence of Apple and Google in that context [18,67].

### Possible Explanations for Low Uptake Rates

At the time of writing this manuscript, over half a year after the launch of the respective contact tracing apps, app developers and authorities were facing lower user rates than initially expected [8,9]. This constitutes some tension with other government measures that also limited people’s freedom but were readily accepted; for example, the majority of people in Germany and Austria supported compulsory face mask use a few months later [12,68,69].

One reason for this might be that the early perception as a surveillance tool for authorities left many people reluctant to use contact tracing apps. The observed tendency to put these contact tracing apps into a broader context of technological surveillance tools and privacy concerns might have triggered general concerns of privacy that are known to be particularly important in the German-speaking areas [70], and the same has been reported in the French context [18]. Newspapers reporting the use of contact tracing and tracking apps in Asian countries might have further deepened the impression that these applications are not aligned with democratic principles of individual privacy and freedom since these applications were considered by interview participants and newspapers alike to be not compatible with privacy regulations and democratic principles as understood in the European context.

The broad contextualization of contact tracing apps makes factual information about the app itself (such as it contributes to controlling the pandemic, data is anonymized, and location tracking impossible) less effective, as people tend to use pre-existing concepts from other technologies to avoid cognitive dissonance, which occurs when expectations and performance contradict each other [71]. Thus, even though the performances of the apps are privacy-friendly, those who are generally skeptical towards digital tools and surveillance still expect them to be dangerous, despite the crucial efforts of app developers and policymakers. This points to the underlying and unsolved issues of regulating digital tools, data ownership, and nontransparent use and economization of data [67].

Likewise, Barth and Jong [72] suggested that the privacy paradox, in which people share some information willingly but are reluctant to share information with others, was relying on risk-benefit assessment (see also [60]). It is plausible that a subset of people considers contact tracing apps to have more privacy-related risks than benefits. Indeed, the currently available contact tracing apps have little personal benefit but promise to serve the common good by contributing to pandemic containment. Proof of effectiveness has been difficult to demonstrate thus far because user rates have been lower than originally suggested [14], so potential benefits could not unfold, and it is challenging to collect relevant data for quality control due to privacy constraints. Showing the benefits of contact tracing apps on an individual and a societal level might increase the uptake, but would need to be reliable, ideally through direct empirical evidence of effectiveness.

### Public Trust and Transparent Communication

Particularly in Austrian interviews, people criticized what they perceived to be the absence of transparent political communication. For example, the public speculation of the president of the first chamber of the Austrian parliament, Wolfgang Sobotka, that the use of the Austrian app may become mandatory seemed to undermine people’s trust. It also influenced participants’ responses in the days following this particular event. Although most Austrian participants trusted the Red Cross, the fact that Red Cross launched the app was often considered less relevant than the function of the app as a surveillance tool for authorities. Trustworthiness was generally a less dominant topic in Swiss and German interviews. In both countries, epidemiologists and app developers were frequently given a voice in newspapers. For instance, newspaper coverage followed discussions of app developers about centralized or decentralized data storage, or it critically reflected on political considerations connected to voluntariness and whether to introduce a legal basis for surveillance measures. These issues were publicly discussed and addressed prior to the release of contact tracing apps in June, and upon their launch, it was already legally binding that apps could not become compulsory and needed to employ decentralized storage systems. This is in sharp contrast with other examples, such as in France, where a centralized storage system was implemented despite privacy concerns [18]. Such discussions prior to launch might have promoted public trust in authorities and could be a possible reason for the higher relative uptake numbers in Germany and Switzerland as compared with those in Austria. This is further supported by our finding that German and Swiss newspapers framed the discussion, particularly the scientific discussion behind app development, from a more international perspective, whereas Austrian newspapers focused on the national issues of the already-released app. In line with our findings, recent ethical inquiries have also identified public trust as the most critical element for the uptake of contact tracing apps [18,40,73-75].

### Conflation of Different Mobile Phone–Based Tracing and Tracking Apps

Newspapers and interview participants alike often conflated different tracking applications (anonymous geolocation or Wi-Fi data population analysis vs. real-time personal GPS tracking versus Bluetooth-based proximity tracing). This seems to have confused people and may have solidified concerns surrounding the possibility of ubiquitous surveillance because instances where de-identified information was collected were interpreted as personalized tracking. These uncertainties and misconceptions seemed to be associated with uneasiness and general skepticism. These findings highlight the difficult task for governments to communicate about the apps they plan to introduce transparently, clearly, and simply, while including sufficient information to allow people to distinguish between different applications [76,77].

### Limitations

This study did not assess the exact prevalence of specific views on, or experiences with, contact tracing apps in the general population in the three countries or regions examined. Instead, we sought to explore how people describe their practices regarding contract tracing apps where they have had personal experience, and their normative, factual, and emotional reference points when discussing the design, utility, and effect of digital contact tracing. To contextualize the data obtained from the large-scale interview study, we also carried out an analysis of mainstream news media in the three countries or regions that was limited by the fact that newspapers were the only format examined and other mass media (such as television, radio, and social media) were excluded. No reliability testing was conducted because we interpreted the data relationally and qualitatively, looking for relationships rather than statistical significance. As such, this article provides context-specific insights into digital tracking tools during a specific period in the COVID-19 pandemic. Even though we sought a balanced demographic distribution, the final sample of interview participants is slightly skewed towards people with higher educational levels (more than 10 years), those who are under 70 years of age, and those in the Austrian sample living in urban areas. However, because this is a qualitative analysis not aiming for statistical representativeness, every view was considered independent of how many participants had stated this view. Given that several interviewees supported every view we report and that we were able to draw what in the grounded theory approach is called a “middle-range theory,” we conclude that we have reached satisfying theoretical saturation [78]. Although we did report when issues were mentioned particularly frequently or particularly rarely, any quantification in terms of how many citizens support these views is subject to further quantitative inquiries, such as follow-up surveys, which go beyond the scope of this paper. In the Swiss context, only the German-speaking part is covered both in terms of newspaper coverage and interview participants. Since there are considerable cultural differences between Swiss language regions [79], our findings are limited to the German-speaking part of Switzerland.

### Conclusions

Newspapers from and people living in German-speaking countries perceived digital contact tracing apps as surveillance tools popularized by authorities during the first wave of the COVID-19 pandemic in April 2020, attributing a broad range of interpretations, evaluations, and impact to this perception. Even though privacy was a common concern among participants, many also framed surveillance in a positive way and saw contact tracing apps as tools that could benefit society in containing the viral spread. Others saw the potential for personal and societal benefit, affording users better control over their exposure risk. Voluntariness and trustworthiness were most frequently discussed by Austrian participants, in line with Austrian newspaper coverage and political discussions at that time. The early launch of the Austrian Stopp Corona app has, on the one hand, made the topic more immediately relevant for both study participants and mass media. On the other hand, ongoing debates about the voluntariness and the use of apps for checking individual compliance through the authorities might have caused more severe and lasting rejections in Austria than in Germany or Switzerland, where the relative uptake of the apps was slightly higher.

Although communication from authorities and app developers shapes peoples’ early perceptions of contact tracing apps, their previous experiences and expectations with authorities and digital tools also play an important role. Thus, when authorities plan to implement new digital tools and practices in the future, they should be very clear in communicating the objectives, the contribution of this technology, and how it differs from other, possibly similar, tools that may be problematic or may have been previously used in a problematic way. Similarly, even in cases where there is a pressing need for the use of new tools, it is important to address publicly and solve ethical, legal, and social issues related to such technologies prior to their launch—existing concerns related to new technologies need to be addressed proactively. Finally, to overcome the privacy paradox, the effectiveness of contact tracing apps needs to be evaluated and communicated.

## Appendix

Multimedia Appendix 1 Qualitative interview guide (SolPan; translated German version, April 2020).

Multimedia Appendix 2 Codebook used for newspaper content analysis.

Multimedia Appendix 3 Supplementary tables of newspaper content analysis.

Multimedia Appendix 4 Country-specific analysis of frames portrayed in newspaper coverage over time.

## Abbreviations

DP-3T: Decentralized Privacy-Preserving Proximity Tracing
mHealth: mobile health
PEPP-PT: Pan-European Privacy-Preserving Proximity Tracing
SolPan: Solidarity in Times of a Pandemic
