# Supplementary material for: Early Perceptions of COVID-19 Contact Tracing Apps in German-Speaking Countries: Comparative Mixed Methods Study
Source: J Med Internet Res. 2021 Feb 8;23(2):e25525. doi: 10.2196/25525 (PMC7872326; doi:10.2196/25525)
Supplement: Multimedia Appendix 1 [file jmir_v23i2e25525_app1.docx]

**Multimedia Appendix 1**

**SolPan interview guide (translated German version, April 2020)**

1. **Can you tell me about when you first heard about Corona**?
   1. What were your thoughts about Corona then?
   2. Did you discuss Corona with anyone?
   3. Did you do anything in particular after you had first heard about Corona?
2. **How has Corona affected your (/family´s) life? What is an average day for you like now?**
   1. What changes, if any, have you made in the way you go about your life? Why? (e.g. concerning work, childcare, shopping (including grocery choices and supporting local business), health care, recreation and leisure, interactions with other people, etc.)
   2. Do you know anyone that is or could be particularly affected by Corona?
   3. If you (/family) get sick (not just with Corona), would you feel confident that you will receive adequate care?
   4. What has been the greatest challenge for you (/your family) in the past few weeks?
3. **How do you feel about the response of authorities to Corona?**
   1. Could they have done anything differently? (e.g. to contain Corona, to support people and businesses, to protect health care workers, coordination of efforts, etc)
   2. What measures do you take to protect yourself or others? Why?
   3. What measures do you not take, although these measures may be recommended, or even compulsory? Why not?
   4. Some authorities are considering using our cell phones to track our location and thus contain the spread of the virus. This would mean that the authorities might know where you are. Have you heard of these ideas, and if so, what do you think about them? With whom did you talk about your feelings?
4. **How do you feel about the response of the wider society to Corona?**
   1. How do you feel about the reaction of industry and business to Corona? What could they have done differently?
   2. What local initiatives are you aware of that support people during Corona? (e.g. food pick-ups). How did you learn about these? Do you know anyone who takes part in these initiatives?
   3. What kind of behavior have you experienced in public that has been influenced by Corona? What conversations and discussions have these generated?
   4. What kind of behaviors have you seen or heard about that surprised you? Why did this surprise you?
5. **Where do you get information about Corona?**
   1. What information sources do you trust the most/least regarding Corona? Why?
   2. What images or stories have stuck with you most?
6. **What kind of long-lasting changes do you expect to come out of this, and how do you feel about them?**
   1. **How do you expect your life to change?** (e.g. interactions with others, travel for tourism and work, working arrangements, increased pay for certain professions)

**To conclude, I would like to ask you a few quick questions about yourself.**

**All information is anonymous, of course.**

1. What year were you born in?
2. What is your gender?
3. How many people currently live in your household including yourself? *[If asked: By “household” we mean a group of people who usually sleep under the same roof.]*
4. *If not a single household:* How many of those people are under the age of 12?
5. What village or city do you live in?
6. Are you currently in employment?
7. If yes: What best describes your situation: are you employed on a long-term contract, on a short-term contract, or are you self-employed?
8. If no: Were you in employment *shortly before* the Corona crisis?
9. If yes: What best describes your most recent employment: were you employed on a long-term contract, on a short-term contract, or were you self-employed?
10. If no: What best describes your situation: are you in education, looking for work, or are you retired?
11. What is your highest educational degree?
12. Considering all members in your household jointly, what would you guess is the income that is at your household’s disposal after taxes: would you say it is above or below 1400 EUR (4000 CHF) per month? If above: Would you say it is above or below 3000 EUR (7000 CHF)?
