# Supplementary material for: Early Perceptions of COVID-19 Contact Tracing Apps in German-Speaking Countries: Comparative Mixed Methods Study
Source: J Med Internet Res. 2021 Feb 8;23(2):e25525. doi: 10.2196/25525 (PMC7872326; doi:10.2196/25525)
Supplement: Multimedia Appendix 2 [file jmir_v23i2e25525_app2.docx]

**Multimedia Appendix 2**

# **Codebook Newspaper Content Analysis on tracking & tracing applications during COVID-19 lockdown**

Adaptions after coder training in green color:

- No coding of ethical issues
- Only code stakeholders that are directly or indirectly cited in the text

## Article ID

## Title

## Country of origin

- 1. Switzerland
  2. Germany
  3. Austria

## Date of publication *– as indicated in factiva database*

## Article length (words*) – as indicated factiva database*

## Medium (newspaper)

*please use the indicated number of the best fitting category*

1. Neue Zürcher Zeitung
2. Tages Anzeiger
3. Neue Luzerner Zeitung
4. Blick
5. Süddeutsche Zeitung
6. Die Welt
7. taz - die tageszeitung
8. BILD
9. Frankfurter Allgemeine Zeitung
10. Kurier (Austria, German Language)
11. Der Standard (Austria, German Language)
12. Die Presse (Austria, German Language)
13. Krone.at (German Language)

## Level of how tracing/tracking applications were mentioned

X Topic of tracing/tracking applications in COVID-19 pandemic not mentioned 🡪 STOP DATA COLLECTION

- 1. Main topic of the article *(covered in the title OR lead text)*
  2. Mentioned as one of several topics *(at least one topic-related paragraph, but other topics covered as well AND topic not explicitly mentioned in the title or lead text)*
  3. Mentioned as a side note (*less than one paragraph*)

## Country reference

*please use the indicated number of the best fitting category*

- 1. Only in-country (D/A/CH)
  2. In-country in comparison with other countries
  3. Other European countries
  4. Other Asian countries
  5. International (mix of various countries worldwide)
  6. Other

1. Unclear

## Comment country reference *(string, please comment on what countries are mentioned)*

## Summary of content: *Main topic(s) of the article (string, please summarise the most important topic-relevant points of the article)*

## Main topic of the article

*Please choose the best fitting category; the MAIN topic is the one* ***mentioned in the title*** *and specified in the lead text.*

x Tracking/tracing technology not the main focus, a completely other topic is the main topic

- 1. GPS tracking, Bewegungsrückverfolgung auf Populationsebene
  2. GPS tracking, Bewegungsrückverfolung auf individueller Ebene (surveillance)
  3. “Datenspende” App (RKI)
  4. Development of tracing apps (process of development, announcement about app introduction, etc.)
  5. Functioning of tracing apps (explanations on how they work)
  6. Centralized vs decentralized data storage (nur codieren wenn es explizit darum geht)
  7. Legal/ethical issues regarding tracing apps

1. Other relevant topics *(please specify in column J)*

## Other article topics (I)

*optional, only if another of the topics below is coming up in the text. Must cover min. one paragraph*

- 1. GPS tracking, movement tracing at population level
  2. GPS tracking, Movement tracing at individual level (surveillance)
  3. “Data donation” app (RKI)
  4. Development of tracing apps (process of development, announcement about app introduction, etc.)
  5. Functioning of tracing apps (explanations on how they work)
  6. Centralized vs decentralized data storage (nur codieren wenn es explizit darum geht)
  7. Legal/ethical issues regarding tracing apps

1. Other relevant topics *(please specify in column J)*

## Other article topics (II)

*optional, only if another of the topics below is coming up in the text. Must cover min. one paragraph.*

- 1. GPS tracking, movement tracing at population level
  2. GPS tracking, Movement tracing at individual level (surveillance)
  3. “Data donation” app (RKI)
  4. Development of tracing apps (process of development, announcement about app introduction, etc.)
  5. Functioning of tracing apps (explanations on how they work)
  6. Centralized vs decentralized data storage (nur codieren wenn es explizit darum geht)
  7. Legal/ethical issues regarding tracing apps

1. Other relevant topics *(please specify in column J)*

## Other article topic (III)

*optional, only if another of the topics below is coming up in the text. Must cover min. one paragraph.*

*IF MORE THAN 4 topics are coming up, please state the rest as a comment in column J*

- 1. GPS tracking, movement tracing at population level
  2. GPS tracking, Movement tracing at individual level (surveillance)
  3. “Data donation” app (RKI)
  4. Development of tracing apps (process of development, announcement about app introduction, etc.)
  5. Functioning of tracing apps (explanations on how they work)
  6. Centralized vs decentralized data storage (nur codieren wenn es explizit darum geht)
  7. Legal/ethical issues regarding tracing apps

1. Other relevant topics *(please specify in column J)*

## Evaluation of application

*please be aware of the “special” numbering!*

*Code 3 if there are both positive and negative arguments. Code 1 or 5 if positive/negative evaluations overweight*

*If several topics are covered, please code evaluation of the main topic*

- 1. Positive - only or for the most part positive arguments/benefits

1. Ambivalent – both positive (benefit) and negative (risks) arguments mentioned
2. Negative - only or for the most part negative arguments/risks
3. No judgment

## Stakeholders cited

*code each group only once, even if e.g. several politicians are cited – but write all their names in the comment section in column Y and mark all cited stakeholders blue in the word document*

*Please only code stakeholders if they are cited directly or indirectly; being mentioned alone does not qualify*

1. Governmental actors / politicians / parliament
2. Non-governmental actors *(e.g. Red Cross, NGOs, churches, etc.)*
3. Scientific and medical experts *(epidemiologists, virologists, data scientists, physicians…)*
4. Legal experts *(data protection officers, lawyers, etc.)*
5. Experts from humanities/social sciences/ethics etc.
6. Celebrities / VIPs *(famous people, e.g. Bill Gates, Angelina Jolie, etc.)*
7. Civil society *(citizens, testimonials from “ordinary” people, letters to the editor)*
8. Private companies (*e.g. tech companies)*
9. Other (please specify)

## Comment on stakeholders cited

*please list the names and functions of all stakeholders coded*

## General coder comments

*please state here any questions and uncertainties, or things that you find particularly interesting in that article*
