# Supplementary material for: Early Perceptions of COVID-19 Contact Tracing Apps in German-Speaking Countries: Comparative Mixed Methods Study
Source: J Med Internet Res. 2021 Feb 8;23(2):e25525. doi: 10.2196/25525 (PMC7872326; doi:10.2196/25525)
Supplement: Multimedia Appendix 3 [file jmir_v23i2e25525_app3.docx]

**Multimedia appendix 3**

**Reporting of additional data from newspaper content analysis**

Table S1: Distribution of importance of digital track & trace applications within each article.

| **Importance of digital track & trace applications within the article** | **Germany** | **Austria** | **Switzerland** |
| --- | --- | --- | --- |
| Main topic of article (in title/lead) | 39 | 43 | 36 |
| Mentioned as one of several topics | 13 | 11 | 17 |
| Mentioned as a side note (less than one paragraph) | 15 | 8 | 12 |
| Total included | 67 | 62 | 65 |

Table S2: Length of articles.

| **Length of articles** | **Germany** | | **Austria** | | **Switzerland** | |
| --- | --- | --- | --- | --- | --- | --- |
|  | **all** | **main topic only** | **all** | **main topic only** | **all** | **main topic only** |
| Short notices (<200 words) | 5 | 4 | 3 | 3 | 4 | 1 |
| News (200-800 words) | 27 | 17 | 46 | 33 | 38 | 26 |
| In-depth reports (>800 words) | 35 | 18 | 13 | 7 | 23 | 9 |
