# Supplementary material for: Early Perceptions of COVID-19 Contact Tracing Apps in German-Speaking Countries: Comparative Mixed Methods Study
Source: J Med Internet Res. 2021 Feb 8;23(2):e25525. doi: 10.2196/25525 (PMC7872326; doi:10.2196/25525)
Supplement: Multimedia Appendix 4 [file jmir_v23i2e25525_app4.docx]

## Multimedia appendix 4

**Newspaper content analysis: Identification of key events and frames**

### Germany

##### Geolocation or wifi-based tracking (15.03.20 – 09.04.20)

- - In mid-March, articles focussed mainly on tracking technologies based on geolocation or wifi data, conflating anonymous population tracking with individual surveillance as performed in Asian countries. Evaluation ambivalent, data protection issues mentioned
  - **25 March 2020: Political discussion about the inclusion of tracking/surveillance measure into the new German Epidemic Law**; concern about data protection and quick change of plans in policy (Jens Spahn) *🡪 incident in following articles often used to introduce articles about tracking/tracing apps; emphasizing surveillance concerns indirectly*
  - After 9 April, no more articles that focused mainly on tracking apps

##### RKI “Datenspende” app (15.03.20 – 28.04.20)

- - “Datenspende” initiative presented as a side note on several occasions in SDZ in March and beginning of April (used for research purposes only, not for population tracing)
  - 9 April: launch; presented in taz & SDZ with negative evaluation 🡪 data protection and transparency issues mentioned
  - Presented again in taz & SDZ on 27/28 April 🡪 still negatively evaluated, but in a broader context and related to the “other” tracing app that might come soon (collecting data in the COVID-19 crisis – fear of exploitation through the state – “transparent citizen” due to technological surveillance more general)

##### Contact tracing app (31.03.20 – 06.05.20)

- - **Phase 1: 31.03. – 02.04.2020: Positive introduction as an option**The option of a Bluetooth based, privacy-friendly tracing app is presented for the first time: several articles in taz/SDZ/Welt, rather positive evaluation, presented as a way to use apps for contact tracing while protecting privacy and data and presentation of the PEPP-PT initiative. Voluntariness is mentioned as another important aspect.
  - **Phase 2: 04.04 – 22.04.2020: A rather negative discussion about international applications**
    An international perspective is taken, rather negative evaluations, data protection, and privacy issues often mentioned; also the question about voluntariness – criticism about the benefits of an app that won’t be used by enough citizens
  - **21.04.20: Eclat in PEPP-PT initiative**Several PEPP-PT members left the initiative, criticizing lack of transparency (open source not a requirement) and data safety issues (centralized instead of decentralized data storage) 🡪 Discussion about surveillance and data protection requirements
  - **27.04.20: German government decides on a decentralized approach**

Positive evaluation: privacy

Critique due to the long duration of app development

### Austria

##### Launch of Stop Corona App & discussion about A1 mobile GPS data for population tracking

- - On 24.03.2020 launch “Stop Corona App” by Red Cross Austria
    - evaluation positive/ambivalent. At the same time, discussions about GPS-tracking (negatively evaluated) 🡪 connections between Corona App and GPS-tracking are made
    - The political dimension of the Stop Corona App: make the “private” app a governmental one? 🡪 discussion on how the use of this could be extended to track people´s whereabouts
      🡪 political discussions seem nontransparent, a lot of speculations, e.g. AT008
    - Speculations about what such apps could be used for 🡪 allow entrance only with tracing app; track people’s whereabouts; analysis of personal data through government
      Trust issues (AT034, 10.4.20)
- **04.04.2020: Key event – Wolfgang Sobotka, President of the Austrian national council, publicly speculates about making the *Stopp Corona app* mandatory**
  - The unveiling of political discussions about making app mandatory, including key chains for those without smartphones 🡪 Causes critical coverage and a public outcry based on civil rights and privacy
  - Politicians take back obligation considerations immediately and frame the *Stopp Corona* *app* as a voluntary element that should support and enable easing of restrictions (5.-6.4.20)
    🡪 despite this, coverage continues to critically examine mandatory apps; the issue of voluntariness continues to be represented in coverage throughout the whole month of April

##### Policy-oriented coverage in mid-April

- 09.04.2020: A new function of Stopp Corona App: automatic handshake (reported but no key event that triggered important coverage)
- 14.04.2020: Reopening of certain stores, first relaxation of restrictions; newspapers report about the possible positive role the *Stopp Corona App* could have in this (starting 11.04.20)

##### Starting to take an international perspective (15.04.2020 – 28.04.2020)

- Comparison of *Stopp Corona App* with solutions proposed in other countries (e.g. Germany, EU, Israel, Scandinavia)

##### 04.05.2020: Key event – the chancellor´s counselor Mei-Pochtler publicly proposes apps that work “at the edge of democracy”

- Causes important negative coverage

### Switzerland

##### Geolocation or wifi-based tracking applications (17 March – 8 April 2020)

- March: anonymous data analysis of GPS-based population tracking – positively evaluated as a useful tool to anonymously follow mobility patterns of Swiss residents during the lockdown
- Whole period: distinction to personalized tracking in China and other Asian countries, which are critically examined (negative evaluation); framing as an application that is not conceivable in Switzerland
  - - Coverage about that topic not daily, but regularly every couple of days

##### Proximity tracing introduced and framed in several stages during April 2020:

- **31 March – 14 April 2020: Introducing proximity tracing, options, and stages of development**

This cluster starts with an interview with epidemiologist Marcel Salathé in NZZ (31 March 2020)

- - - Newspaper coverage accompanies the development of tracing apps, including the PEPP-PT project
    - A cluster of articles about that topic on 3 April 2020 in three newspapers
    - scientists/epidemiologists and public health officers are the predominant stakeholders. Predominantly positive evaluation but issues such as data protection, privacy, feasibility for risk groups are discussed
    - Proximity tracing app as a way to escape lockdown
- **15 April: Key event: Federal Council announced the first step of relaxation (as of 28 April)**
  - - Coverage focuses on the use of tracing apps after lockdown as support for contact tracing teams
    - The government is involved in app development
    - Focus on public trust that is necessary for the success of proximity tracing apps (15-24 April)
- **18 April: Key event: Swiss researcher M. Salathé leaves PEPP-PT consortium**
  - - Negative assessment of PEPP-PT initiative, critique for untransparent communication (18/20 April)
    - M. Salathé positively framed; his decentralized data storage solution is embraced and positively framed in subsequent coverage (22 April)
- **22 April: framing as a support tool for containment and practical concerns regarding proximity tracing app (ambivalent evaluation)**
  - - Now that the app is almost ready (with decentralized storage), newspapers pick up more practical concerns: Concern that the app could be mandatory (23 April, Blick & TagesAnzeiger / 5 May TagesAnzeiger) and the importance of public trust (24 April)
    - 2 May: citizens support tracing apps but members of parliament are often skeptical (surveys)
    - Framing of benefits: How app could be embedded in future containment & contact tracing strategies
    - Anticipation that the app might be launched within days (6 May)
